# Proteomic surveillance of retinal autoantigens in endogenous uveitis: implication of esterase D and brain-type creatine kinase as novel autoantigens

**Published:** 2008-06-12

**Authors:** Yoko Okunuki, Yoshihiko Usui, Takeshi Kezuka, Takaaki Hattori, Kayo Masuko, Hiroshi Nakamura, Kazuo Yudoh, Hiroshi Goto, Masahiko Usui, Kusuki Nishioka, Tomohiro Kato, Masaru Takeuchi

**Affiliations:** 1 Department of Ophthalmology, Tokyo Medical University, Tokyo, Japan; 2 Department of Bioregulation and Proteomics, Institute of Medical Science, St. Marianna University School of Medicine, Kawasaki, Japan

## Abstract

**Purpose:**

Various retinal proteins are newly exposed to immune system in a process of tissue destructive endogenous uveitis. Some of such proteins could be autoantigens that extend the ocular inflammation in human endogenous uveitis. In this study, we aimed to investigate the possibility of such spreading of autoantigens in endogenous uveoretinitis using a proteomic approach.

**Methods:**

Experimental autoimmune uveoretinitis (EAU) was induced in mice by inoculation with a peptide consisting of amino acids 1–20 (GPTHLFQPSLVLDMAKVLLP) of interphotoreceptor retinoid binding protein (IRBP). Six weeks after immunization, the presence of autoantibodies against the retinal proteins in mice with EAU were examined by two-dimensional electrophoresis followed by western blotting (2D-WB). Retinal proteins targeted by the autoantibodies were identified by mass spectrometry (MS) and their autoantigenicity in patients with endogenous uveitis, such as Behcet’s disease (BD, n=36), Vogt-Koyanagi-Harada disease (VKH, n=16), and sarcoidosis (n=17) were examined by enzyme-linked immunosorbent assay.

**Results:**

Six new candidate autoantigens, which were detected in mice with EAU using 2D-WD were identified by MS as β-actin, esterase D (EsteD), tubulin β-2, brain-type creatine kinase (BB-CK), voltage-dependent anion-selective channel protein, and aspartate aminotransferase. Among the patients with endogenous uveitis, 25% of BD and 25% of VKH patients were positive for anti-EsteD antibody, and 25% of VKH and 38.4% of sarcoidosis patients were positive for anti-BB-CK antibody.

**Conclusions:**

Autoantibodies to EsteD and BB-CK produced in EAU-induced mice were also detected in some endogenous uveitis patients, suggesting that these proteins might be autoantigens spreading in a process of endogenous uveoretinitis.

## Introduction

Endogenous uveitis, represented by Behcet’s disease (BD), Vogt-Koyanagi-Harada disease (VKH), and sarcoidosis, is a sight-threatening ocular inflammation. While the pathogenesis of endogenous uveitis is considered to be related to autoimmunity [1-4], the underlying mechanism is not fully understood. Previous reports have suggested the involvement of several autoantigens in the pathogenesis of endogenous uveitis. For example, autoimmunity against ocular-specific antigens [5-7], heat-shock protein (HSP) family [4,8], and ubiquitously expressed proteins [9,10] were reported in BD, and autoimmunity against the melanocyte-associated antigens [11-13] were reported in VKH. However, some of these antigens show autoantigenicity in more than one endogenous uveitis, suggesting the existence of autoantigens that are not disease-specific. For example, autoimmunity to HSP70 was reported in both BD and sarcoidosis [3,4], and autoimmunity to S-antigen was reported in BD, sarcoidosis, and VKH [3]. Although VKH is regarded as a melanocyte specific autoimmune disease, there are reports of autoantigens besides melanocyte specific proteins [3-14]. Therefore, it is considerable that a number of autoantigens are involved in patients with endogenous uveitis.

To explain the phenomena that common antigens are targeted in several endogenous uveitis, we hypothesized that uveitis-mediated destruction of ocular tissue leads to the exposure of ocular antigens to the immune system, which in turn induces the production of antibodies against some of these antigens secondary to the ocular damage. Even though such secondary induced antigens may not participate in the disease-specific pathogenesis in endogenous uveitis, they may modulate different endogenous uveitis in the same manner. Thus, identifying the profile of secondary autoantigens will be of primary importance to understand the pathogenesis of endogenous uveitis. For that purpose, we applied a proteomic approach using two-dimensional gel electrophoresis and western blotting (2D-WB). Both procedures have been shown by us and others to be useful in identifying circulating autoantibodies in autoimmune diseases [15,16].

In this study, we first used the interphotoreceptor retinoid protein (IRBP)-induced murine model of autoimmune uveitis (experimental autoimmune uveoretinitis, EAU) for detecting autoantigens exposed as a result of uveitis-mediated tissue destruction. We screened circulating autoantibodies in mice with EAU against the retinal proteins using 2D-WB and subsequently identified the proteins targeted by the autoantibodies using mass spectrometry (MS). We then evaluated the autoantigenicity of these identified retinal proteins in patients with endogenous uveitis (BD, VKH, and sarcoidosis).

## Methods

### Patients

Serum samples were obtained from 36 BD patients with uveitis (31 men, 5 women; average age 36.9±10.5 years), 16 patients with VKH (12 men, 4 women; average age 39.2±12.6 years), and 17 patients with sarcoidosis (4 men, 13 women; average age 59.6±17.6 years). All patients were followed up by the outpatient clinic of the Department of Ophthalmology in Tokyo Medical University Hospital, Tokyo, Japan. Participants were considered to have BD if their symptoms fulfilled the diagnostic criteria of the Bechet’s Disease Research Committee of Japan [17]. At the time of sample collection, BD patients were determined to be in the active phase of the disease if they presented with one of the following indicators: iridocyclitis with hypopyon, dense vitreous opacity, obscure fundi observation, and retinal exudates with hemorrhages. All VKH patients showed typical exudative retinal detachment confirmed by fluorescein angiography. Sera were collected in these patients before aggressive systemic corticosteroid therapy was begun. Three of the VKH patients were examined when they were experiencing a recurrent phase of ocular inflammation. Ocular sarcoidosis was diagnosed based on the criteria established by the Japanese Committee for Diffuse Lung Disease for the systemic sarcoidosis [18,19]. In total, 64 healthy volunteers (35 men, 29 women), who were age- and sex-matched for each uveitis group, were recruited as healthy controls (HC). The investigation was conducted in accordance to the tenets of the Declaration of Helsinki.

### Animals and reagents

C57BL/6 female mice, aged 6 to 9 weeks, were obtained from Sankyo Laboratory Service Corp (Tokyo, Japan). Animals were handled in accordance with the Association for the Vision and Ophthalmology statement for the use of Animals in Ophthalmic and Vision research. Horseradish peroxidase (HRP)-conjugated rabbit antimouse IgG and goat antihuman IgG were obtained from Zymed Laboratories (San Francisco, CA). Human IRBP peptide 1–20 (hIRBP-p) was synthesized by conventional solid-phase techniques on a peptide synthesizer (Takara Bio Inc., Shiga, Japan). Complete Freund’s adjuvant (CFA) and Mycobacterium tuberculosis H37Ra were purchased from Difco (Detroit, MI), Bordetella pertussis (PTX) was from Sigma-Aldrich Corp (St. Louis, MO), purified human β-actin (bAct) was from Cytoskeleton (Denver, CO), recombinant human esterase D (EsteD) was from GenWay Biotech (San Diego, CA), brain-type creatine phosphokinase (BB-CK) was from Biogenesis (Poole, England), and pETBlue-2 and Turner (DE3) pLacIcompetent cell were from Novagen (Darmstadt, Germany).

### Induction and scoring of experimental autoimmune uveoretinitis

Induction of EAU in C57BL/6 mice and scoring of ocular inflammation were performed as previously described [19]. Briefly, mice were immunized subcutaneously in the occipital region with 200 μg hIRBP-p in 200 μl emulsion in CFA that had been supplemented with H37Ra to 5 mg/ml. Next, 1 μg PTX was given intraperitoneally as an additional adjuvant. On day 18 after immunization, ocular inflammation was clinically evaluated on a scale of 0 to 4 according to the previous report [20]. Briefly, minimal vasculitis with few very small lesions were scored as 0.5; mild vasculitis with 5 or less small focal chorioretinal lesions as 1; multiple (more than 5) chorioretinal lesions or severe vasculitis as 2; pattern of linear choreioretinal lesions, subretinal neovascularization, and haemorrhages as 3; and large retinal detachment and retinal atrophy as 4. The sera of the mice scoring over 1.5 were collected six weeks after immunization and used as the primary antibody in 2D-WB. Control mice were immunized with only CFA and PTX and then processed in the same way as the EAU mice.

### Protein extract preparation

Mice were killed by cervical vertebra dislocation, and then retina from ten normal C57BL/6 female mice were separated immediately after enucleation and whole-tissue lysates were prepared by the freeze–thaw method using a lysis buffer that contained 7 M urea, 2 M thiourea, and 4% CHAPS [21]. The supernatant of each sample was collected, mixed together, and then stored at −80 °C until used.

### Two-dimensional western blotting

Two-dimensional electrophoresis was performed as described previously [21,22]. Briefly, 100 μg extracted protein was applied on 11 cm linear Immobiline Drystrips, pH 3–10 (GE Healthcare Bio-Science Corp., Piscataway, NJ) and rehydrated at room temperature for 10 h. Isoelectric focusing (IEF) was performed using Ettan IPGphorII (GE Healthcare Bio-Science Corp.) for 12 h. After IEF, the strips were equilibrated for 30 min in a denaturation buffer that contained 6 M urea, 2*%* sodium dodecyl sulfate (SDS), 50 mM Tris-HCl, 30% glycerol, and 100 mg/10 ml dithiothreitol (DTT). Strips were then embedded onto 12.5% sodium dodecyl sulfate (SDS)-polyacrylamide gels, and gels were run for 2.5 h. After electrophoresis, the separated proteins on the gels were transferred onto nitrocellulose membranes. For 2D-WB, membranes were blocked in phosphate-buffered saline with 0.1% Tween20 (PBST) containing 0.5% bovine serum albumin (BSA) for 30 min and washed three times with PBST (10 min each time). Membranes were incubated for 1 h in 1:500 times diluted murine serum samples in 0.5% BSA-PBST. Next, membranes were washed with PBST before they were incubated for 1 h with 1:2000 diluted HRP rabbit antimouse IgG. Bound antibodies were visualized using diaminobenzidine (DAB; Dojindo Laboratories, Kumamoto, Japan).

### Protein identification

Protein spots, which corresponded to the EAU-positive spots on the 2D-WB, were recovered from a 2D gel stained with SYPRO Ruby (Molecular Probes, Eugene, OR) and digested in gel with trypsin. The digested peptides were extracted from the gel pieces using trifluroacetic acid and acetonitrile. After centrifugation, the supernatant was recovered. After three more cycles of this extraction, the supernatant was filtered and concentrated down to 10 μl in an evaporator [23,24]. Masses of the digested peptides were determined using a matrix-assisted laser desorption/ionization-time of flight mass spectrometer (MALDI-TOF MS; Ultraflex, Bruker Daltonics, Germany). A list of the determined peptide masses were subjected to mass fingerprinting by using the MASCOT software program version 2.1.0 (Matrix, Science, London UK), in which the National Center for Biotechnology Information (Bethesda, MD) protein data base was searched [25]. Only those proteins with a Mowse score above statistical significant identity (p<0.05) were accepted as identified ones.

### Recombinant protein

Based on the nucleotide sequence of the human tubulin-β (Tub-b) cDNA, the entire protein-coding region of the Tub-b cDNA from the murine retina was amplified using specific pairs of oligonucleotide primers. The amplified cDNA fragment was subcloned into the plasmid expression vector pETBlue-2, and then the protein was expressed in *Escherichia coli* Turner (DE3) pLacIcompetent cells. Thereby, recombinant Tub-b with a tag of six histidines in its COOH-terminal was produced. The purification of Tub-b was performed as follows by brief modification of the previous report [26]. The bacteria was harvested from a 500 ml culture by centrifugation, lysed in 10 ml of lysozyme solution (0.2 mg/ml lysozyme, 1% NP-40 in PBS), and then sonicated to fragment the bacterial DNA. 0.1 mg DNase and 10 mmol MgCl_2_ were added to the sonicated sample. 25 min after, 20 ml of denaturation buffer was added to make final concentration of 8 M urea, 12 mM 2-mercaptoethanol, and 10 mM imidazol. After centrifugation, the sample was filtered, and then the protein was purified using a histidine-Ni+ affinity column (His Trap HP; GE Healthcare Bioscience Corp.) The sample was applied to the column and eluted with an elution buffer (10 mM-500 mM imidazol, 8 M Urea in PBS, pH 7.6).

### One-dimensional western blotting

One-dimensional (1D)-WB was performed as follows. Each protein (4 μg) was separated by 12.5% SDS–PAGE in a buffer that contained DTT, and then transferred onto nitrocellulose membranes. The serum samples were diluted to 1:200 in 1% BSA-PBST and used for incubation with the membranes for 1 h. Incubation of the membranes with the secondary antibody and treatment with DAB were performed as described in the previous section for 2D-WB.

### Enzyme-linked immunosorbent assay (ELISA)

Ninty-six-well microtiter plates (Thermo Labsystems, Franklin, MA) were coated with 5 ug/ml of EsteD, bAct, or BB-CK at 4 °C for 16h in a carbonate buffer which contained 50 mM sodium carbonate. The plates were washed three times with PBST then incubated in 1% BSA-PBST at room temperature for 1 h. The plates were again washed three times, and 50 μl of 1:200 diluted serum samples were added to each well. The plates were incubated for 2 h, and then washed five times with PBST. Following incubation with 1:2000 HRP-goat anti-human IgG for 1 h, the plates were washed eight times with PBST. Color development was achieved by adding 100 μl of 0.04% o-phenylenediamine as a substrate. The reactivity of the serum sample to each protein was expressed using the arbitrary binding units, calculated according to the following formula: sample (binding units)=(OD sample/[mean OD control sera + 2SD of control sera] × 100), where OD is the optical density. According to the formula, 100 binding units were used as the cutoff point.

IgG1 and IgG2a subclasses of anti-EsteD and anti-BB-CK antibodies were determined using the mouse IgG1 and IgG2a quantitation kits (Bethyl laboratories, Montgomery, TX). Briefly, EsteD or BB-CK was coated onto the microtiter plates as described in the previous section, and serum samples diluted to 1:100 were used for the detection of IgG1 and undiluted samples were used for the detection of IgG2a.

### Statistical analysis

Fisher’s exact probability test was used to compare differences in the prevalence of autoantibodies between the HC and each disease group. Fisher’s exact probability test or Student’s *t*-test was used to compare differences in the clinical parameters or clinical symptoms between the antibody-positive and -negative patient groups.

## Results

### Detection of retinal autoantigens involved in experimental autoimmune uveoretinitis

**Figure 1:**
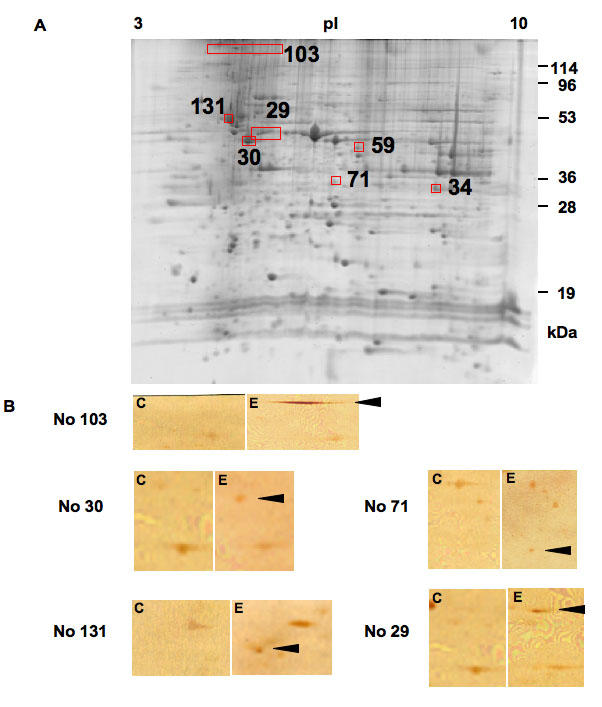
Detection of autoantigens in experimental autoimmune uveoretinitis (EAU). **A:** Shown is a two-dimensional gel image of murine retinal proteins stained with SYPRO Ruby. Approximately 2,000 spots were observed. Among the SYPRO Ruby stained protein spots, the 2D-WB positive spots either in control or EAU were randomly numbered. The numbers and the positions of the seven candidate autoantigens in EAU on a SYPRO Ruby stained gel are shown in panel **A**. The spot numbers of the candidate autoantigens are common between panel **B**, Table 1, and Table 2. **B:** Extracted murine retinal proteins were separated by two-dimensional electrophoresis, transferred onto nitrocellulose membranes. Western blotting was performed using sera from EAU or control mice. Representative membranes reacted with sera from complete Freund's adjuvant-treated control mice are shown in subpanel **C**, and those reacted with sera from EAU mice are shown in subpanel **E**. Each set of **C** and **E** subpanels shows the corresponding area. Arrowheads indicate the position of each candidate autoantigen on the EAU membranes. membranes.

EAU was induced in C57BL/6 mice by immunization with hIRBP-p. Clinical severity was evaluated 18 days after immunization. As a result, approximately 70% of hIRBP-p immunized mice developed ocular inflammation, with the clinical score of more than 1.5. We used sera from ten EAU and ten control mice to comprehensively detect retinal autoantigens by 2D-WB, and we obtained a total of 151 protein spots that reacted positively with any of the tested sera. Though the control and EAU sera showed similar positive pattern on 2D-WB, we detected seven candidate autoantigens (Figure 1A and Table 1) by comparing the positive rate of each spot between the EAU and control by referring to the respective p value. For this purpose, the p value for a protein spot reacting positively to three EAU sera and no control sera was determined to be 0.105, and we chose all the spots as candidate autoantigens whose p values were 0.105 or less. Spot positions of five major candidate autoantigens are shown in Figure 1B.

**Table 1:** Candidate retinal autoantigens detected in experimental autoimmune uveoretinitis by 2D-WB

**Positive rate**			
**Spot number**	**Control**	**EAU**	p value*
103	0/10	10/10	5.4×10-6
30	2/10	6/10	0.084
71	2/10	6/10	0.084
131	0/10	4/10	0.043
29	0/10	3/10	0.105
34	0/10	3/10	0.105
59	0/10	3/10	0.105

We detected seven candidate autoantigens by comparing the positive rate of each spot between the experimental autoimmune uveoretinitis (EAU) and control by referring to the p value of the positive rate. The asterisk indicates Fisher’s exact probability test.

The MALDI-TOF MS and MS/MS analysis were used to identify the seven candidate autoantigens and the results of MS/MS analysis are summarized in Table 2. One spot (number 103), which reacted with 100% of the EAU sera but none of the control sera, was identified as the commonly known retinal binding protein 3 (rbp3). Rbp3 contains the 20 amino acid sequence of the hIRBP-p used for the induction of EAU. Thus, the positive reaction to this protein spot in all the EAU sera was due to the initial immunoresponse against the hIRBP-p used for immunization. It is therefore possible that the remaining six proteins were candidate retinal autoantigens induced as a result of EAU-mediated retinal destruction.

**Table 2:** Identification of candidate retinal antigens in experimental autoimmune uveoretinitis

**Spot** **number**	**Protein name**	**NCBI accession number**	**Calculated MW (kDa)/pI**	**MOWSE score^a^**	**Peptides matched (coverage; %)**	**MS/MS sequence^b^**
103	retinol binding protein 3, interstitial	gi|21729751	134/4.98	208	4 (5%)	IGQSNFFLTVPVSR
30	beta-actin (bAct)	gi|49868	39.4/5.78	83	1 (4%)	SYELPDGQVITIGNER
71	esterase D (EsteD)	gi|55777188	31.8/6.70	52	3 (14%)	SYGQQAASEHGLVVIAPDTSPR
131	tubulin beta-2 (Tub-b)	gi|13542680	50.2/4.79	144	3 (8%)	FPGQLNADLR
29	creatin kinase, brain (BB-CK)	gi|10946574	42.9/5.4	209	3 (11%)	VLTPELYAELR
34	voltage-dependent anion-selective channel protein	gi|10720404	32.5/8.55	39	1 (6%)	WNTDNTLGTEITVEDQLAR
59	aspartate aminotransferase	gi|871422	46.4/6.68	142	4 (10%)	VGGVQSLGGTGALR

^a^Proteins with MOWSE score over statistical significant identity (p<0.05) were accepted as identified. ^b^For the proteins in which several peptides were matched, the peptide identified with the highest MOWSE score is shown.

### Confirmation of immunoreactivity of candidate autoantigens in experimental autoimmune uveoretinitis

**Figure 2:**
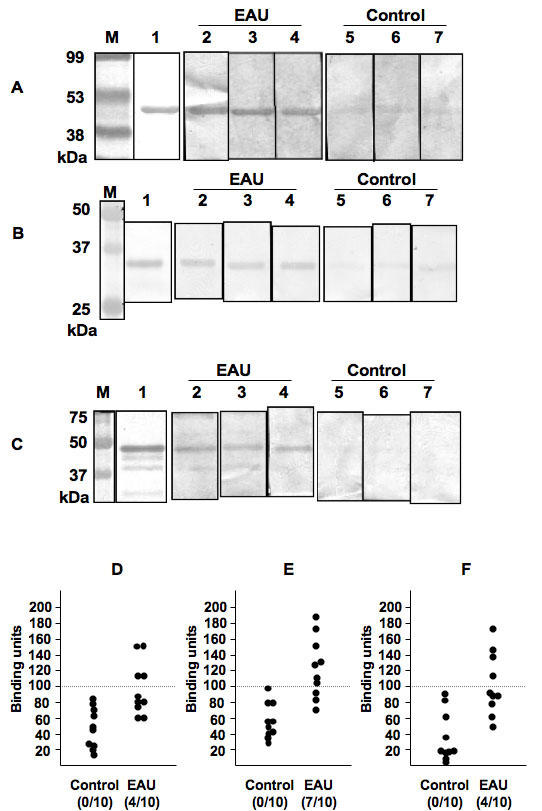
The presence of antibodies to bAct, EsteD, and BB-CK in EAU mice was confirmed by 1D-WB and ELISA. **A-C**: Protein (2 μg) was subjected to 12.5% SDS–PAGE, transferred onto nitrocellulose membranes, then incubated with 1:200 sera from experimental autoimmune uveoretinitis (EAU) or control mice and 1:2000 horse radish peroxide (HRP) antimouse IgG as described in Methods. EAU serum samples were 8/10 positive in bAct (**A**), 6/10 positive in EsteD (**B**), and 4/10 positive in brain-type creatine kinase (BB-CK; **C**). **D-F**: Antibody titer of the sera from the EAU or control mice was determined by ELISA. The antibody titer was calculated as binding units according to the formula shown in Methods. EAU serum samples were 4/10 positive in bAct (**D**), 7/10 positive in EsteD (**E**), and 4/10 positive in BB-CK (**F**). Lane 1 is ponseau S staining, lane 2-4 are membranes incubated with EAU sera, and lane 5-7 are membranes incubated with control sera. In the figure, M represents molecular weight marker.

Among the candidate autoantigens, we chose the four antigens, bAct, EsteD, Tub-b, and BB-CK, for further analysis. We selected bAct, EsteD, and Tub-b because their p values were low (Table 1), and BB-CK because it was a relatively retina- and brain-specific protein. To confirm the aforedescribed results of the 2D-WB, we evaluated autoantibody titers against recombinant bAct, EsteD, Tub-b, and BB-CK in the EAU mice by using 1D-WB and ELISA. 1D-WB analysis showed that 8/10, 6/10, and 4/10 EAU mice were positive for autoantibodies against the antigens of bAct, EsteD, and BB-CK, respectively (Figure 2A,B,C); in contrast, none of the control mice reacted positively with any of these three antigens. Similarly, ELISA analysis showed that 4/10, 7/10, and 4/10 EAU mice were positive for autoantibodies against the antigens of bAct, EsteD, and BB-CK, respectively (Figure 2D,E,F). The results of the 2D-WB, 1D-WB, and ELISA corresponded closely to one another. Similar ELISA results were obtained using another set of control and EAU sera collected six weeks after immunization (data not shown). In addition, when we compared the antibody level against bAct, EsteD, and BB-CK using the murine sera collected three, six, and ten weeks after the immunization, we found that the sera collected six weeks after the immunization had the highest levels of antibodies against these three antigens (data not shown). As Tub-b did not react with the EAU sera in either 1D-WB or ELISA (data not shown), it was not used for any further experiments.

### IgG subclass of anti-EsteD and anti-BB-CK antibodies

**Figure 3:**
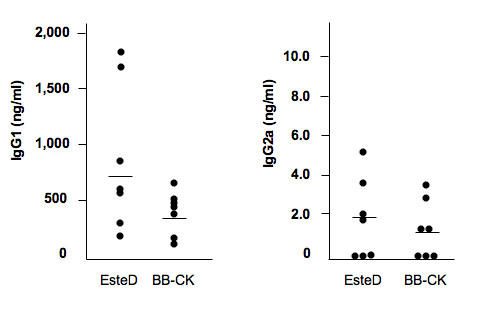
IgG subclass of anti-EsteD and anti- brain-type creatine kinase antibodies in experimental autoimmune uveoretinitis mice were determined by ELISA. Concentrations of anti-EsteD and anti- brain-type creatine kinase (BB-CK) IgG1 antibodies in all tested serum samples of experimental autoimmune uveoretinitis were much higher than those of anti-EsteD and anti-BB-CK IgG2a antibodies. All control serum samples collected from mice immunized without hIRBP-p were negative for both IgG1 and IgG2a.

IgGs are subclassified depending on the types of Th immune responses. Th1-mediated immune response promotes IgG2a production, while Th2-mediated immune response promotes IgG1 production. Thus, we next determined the subclass of the anti-EsteD and the anti-BB-CK antibodies. Interestingly, most of the anti-EsteD and anti-BB-CK antibodies in all the tested EAU serum samples were IgG1, and the amount of IgG2a type of the anti-EsteD and anti-BB-CK antibodies were much lower than their respective IgG1 antibodies (Figure 3). All control serum samples collected from the mice immunized without hIRBP-p were negative for both IgG1 and IgG2a (data not shown).

### Autoantigenicity in human uveitis

As shown in the previous section, bAct, EsteD, and BB-CK were confirmed as retinal autoantigens in the murine model of autoimmune uveitis. Because the amino acid sequence of these three autoantigens are highly conserved in human and mouse (approximately 91–100%), we next examined whether autoantibodies against these antigens could be detected in human endogenous uveitis.

**Figure 4:**
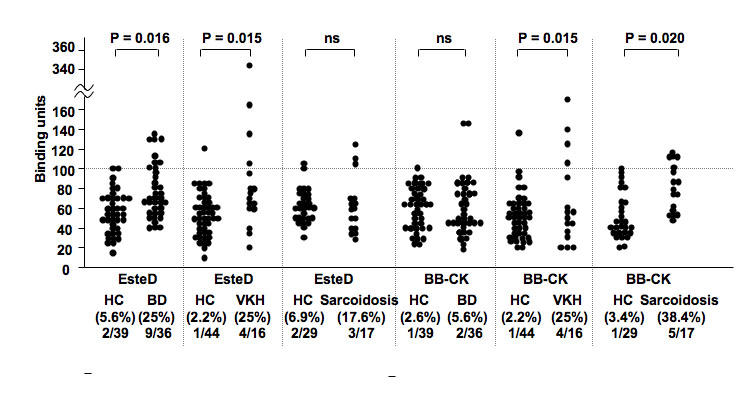
Autoantibodies against the retinal autoantigens detected in experimental autoimmune uveoretinitis were tested using sera from human endogenous uveitis patients. The antibody titer was calculated as binding units according to the formula given in Methods. Statistically significant difference of positive rate between each patient group and healthy control (HC) was detected in Behcet’s disease (BD; p=0.016) and Vogt-Koyanagi-Harada disease (VKH; p=0.015) for anti-EsteD antibody, and in VKH (p=0.015) and sarcoidosis (p=0.036) for anti- brain-type creatine kinase (BB-CK).

We examined the immunoreactivity against bAct, EsteD, and BB-CK by ELISA in 36 BD, 16 VKH, and 17 sarcoidosis patients with uveitis and age- and sex-matched HC. A profile of these patients is given in Table 3. Patients with the medical history of all four symptoms associated with BD (oral aphthae, skin lesion, genital ulcer, and uveitis) were 36.1%. For the VKH group, patients with active ocular inflammation before the starting of aggressive corticosteroid therapy were used, because high dose corticosteroid therapy would dramatically suppress immune response and antibody titers would also be strongly suppressed. As shown in Figure 4, EsteD reacted with the sera from 25% of BD, 25% of VKH, and 17.6% of sarcoidosis patients, and the percentages were significantly higher in BD (p=0.016) and VKH (p=0.015) groups than in the control group. BB-CK reacted with sera from 5.6% of BD, 25% of VKH, and 38.4% of sarcoidosis patients. Percentages were significantly higher in VKH (p=0.015) and sarcoidosis (p=0.020) groups than in the control group. One sample each in BD and sarcoidosis and two samples in VKH were positive for both EsteD and BB-CK. However, bAct did not react positively with any of the uveitis sera with any statistical significance (data not shown).

**Table 3:** Profile of patients whose sera were used for antibodies detection by ELISA

**Parameter**	**BD (n=36)**	**VKH (n=16)**	**Sarcoidosis (n=17)**
Age (year)	36.9±10.5	39.2±12.6	59.6±17.6
Sex (Male:Female)	31:5	8:8	4:13
Active ocular inflammation	52.8%	100%	76.4%
Duration from the onset	4.8±4.1 (year)	8.8±10.5 (day)	6.2±5.3 (yr)
LogVA	-1.18±1.14	-0.43±0.48	-0.36±0.52
Administration of systemic corticosteroid	32.1%	12.5%	5.9%

In the table “duration from the onset” refers to the interval from the onset of the disease to the day when blood was collected.

Next, we compared the clinical parameters of patients between the anti-EsteD antibody and anti-BB-CK antibody positive- and negative-patient groups in each uveitis group. As shown in Table 4, every anti-EsteD antibody positive VKH patient in this group was female. Results from this group were statistically significant compared to the anti-EsteD antibody negative VKH group (p=0.034). In the case of the BD or sarcoidosis patients, there were no statistically relevant correlations between the examined parameters (age, sex, disease duration, visual acuity, activity of ocular inflammation, serum IgG levels, systemic medications at the time of sample collection, presence of HLA-B51, or involvement of systemic diseases) and the presence of either anti-EsteD or anti-BB-CK antibody (results not shown).

## Discussion

We have identified six candidates of retinal autoantigens in EAU, out of which two, EsteD and BB-CK, showed autoantigenicity in both human endogenous uveitis and EAU. EsteD (EC 3.1.1.1) is a 282 amino acid cytoplasmic protein (31.4 kDa) that is ubiquitously expressed and functions as a nonspecific esterase. Our study showed that the murine retina expressed EsteD, and a previous work reported the presence of EsteD in human retinal pigment epithelium [27]. Considering that EsteD is expressed abundantly in other tissues such as liver, kidney, and intestine [28], there is a possibility that the anti-EsteD antibody would be found in other autoimmune diseases. However, our present study is the first to show EsteD is a target of autoimmunity in uveitis. In this study, three out of four anti-EsteD antibody-positive VKH patients were examined when they were experiencing their first episode of ocular inflammation, and one patient was examined when experiencing a recurrent phase. Our results suggest that even though VKH is a melanocyte-specific autoimmune disease, autoimmunity to antigens other than melanocyte-specific proteins are induced from the early period of uveitis, and these autoantibodies may modulate the disease. Several reports about the melanocyte-unrelated antigens in VKH support this idea [3,14]. In the VKH group, all of the anti-EsteD positive patients were female, and the result was statistically significant. However, a larger number of patients are needed to determine that female VKH patients are commonly positive for anti-EsteD antibody.

Another retinal autoantigen detected both in EAU and human uveitis was BB-CK (EC 2.7.3.2). This is a 381 amino acid cytoplasmic enzyme (42.6 kDa) that is abundantly expressed in brain and retina, and its expression in retina has been mentioned in several reports [29,30]. Autoantibody production against BB-CK was also reported in patients with paraneoplastic sensory-dominant neuropathy [31]. Interestingly, autoantigens common to both the paraneoplastic syndrome and endogenous uveitis were reported previously. For example, it was shown that the anti-α-enolase antibody was present both in BD [9] and cancer-associated retinopathy [32-35], a paraneoplastic syndrome that affects visual function. Considering BB-CK is relatively abundant in the retina, the anti-BB-CK antibody may modulate vision-related functions in anti-BB-CK antibody-positive patients, although statistical differences in the clinical parameters were not observed in the present study.

BD, sarcoidosis, and VKH exhibit clinically distinguishable ocular manifestations, and their etiologies and pathogenesis are also different. We did not observe any statistical correlation between the patients positive for these antibodies and the disease parameters. The only distinguishing feature in this study was all anti-EsteD antibody positive patients were female. We suggest that production of anti-EsteD antibody or anti-BB-CK antibody might be unrelated to the type of uveitis—that is, regardless of the classification of uveitis, some of the patients would be positive for these antibodies. It would be worthwhile to examine a greater number of patients to further understand the roles of these autoantibodies in endogenous uveitis.

Some proteins are secondary exposed to immune system in a process of autoimmune disease. Some of such proteins could be autoantigenic and become one of the factors that extend inflammation. Several mechanisms, such as epitope spreading, molecular mimicry, and bystander activation, have been reported in various animal models of autoimmune disease and in some human autoimmune diseases [36,37]. In the present study, the anti-EsteD and anti-BB-CK antibodies were mainly of the IgG1 subclass, production of which is promoted by Th2-type cytokine. However, EAU is considered to be a Th1 disease. Therefore, it is possible that anti-EsteD and anti-BB-CK antibodies were produced as a result of Th2 immune responses promoted in the process of downregulating EAU. However, we can not define immune responses against EsteD or BB-CK in the development of endogenous uveitis with the results described in this study. We plan to study the immunogenicity including antigenicity of these proteins. In this regard, although it is a preliminary experiment, it is worth mentioning here that we have recently immunized C57BL/6 mice and Lewis rats with these two autoantigens; however, we did not observe uveitis in these animals until day 21 after immunization. This result tends to support the idea that any immune response against these two autoantigens is, indeed, a secondary reaction to tissue damage caused by the primary uveitogenic process.

Among the EAU-associated retinal autoantigens found in this study, all but BB-CK are ubiquitously and abundantly expressed proteins. These results suggest that in an underactivated immune system, even ubiquitously expressed antigens would become a target of organ-specific autoimmunity. Considering that none of the previously reported ocular-specific autoantigens in endogenous uveits (such as S-antigen, rhodopsin [38], and recoverin [39]) were detected as candidate retinal autoantigens in the present study, ubiquitously expressed proteins appear to be more immunogenic among antigens exposed to immune system secondarily under tissue-destructive severe uveitis.

In conclusion, our study demonstrated that autoantibodies were produced against several proteins in EAU induced by immunization with an IRBP-derived peptide. Of these, autoantibodies specific for EsteD and BB-CK were also detected in human endogenous uveitis. Therefore, we suggest that EsteD and BB-CK might be autoantigens secondarily generated as a result of uveitis-induced retinal destruction. Our approach of using an animal model of autoimmune disease to comprehensively detect autoantibodies was useful in identifying new autoantigens. We believe that similar approaches will also facilitate the detection of autoantigens in other autoimmune diseases.

## 

**Table 4:** Statistical analysis of clinical parameters between anti-EsteD positive and negative patients of Vogt-Koyanagi-Harada disease

**Parameter**	**P (n=4)**	**N (n=12)**	**p value**
Age (year)	35.8±12.7	42.1±10.3	0.41^a^
Sex (M:F)	0:4	8:4	0.04^b^
HLA-DR4	75% (n=4)	77.8% (n=9)	0.91^b^
LogVA	-0.49±0.61	-0.42±0.21	0.84^a^
IgG (mg/ml)	11.6±2.1	10.1±3.7	0.46^a^

Four anti-EsteD antibody positive Vogt-Koyanagi-Harada (VKH) patients and 12 anti-EsteD antibody negative VKH patients were statistically compared their age, sex, Log visual acuity (VA), and serum concentration of total IgG. For the analysis of HLA-DR4, four anti-EsteD antibody positive and 9 anti-EsteD antibody negative VKH patients were analysed. Age, LogVA, and serum IgG concentration were compared by Student’s t test, and sex and the presence of HLA-DR4 were compared by Fisher’s exact probability test. Anti-EsteD antibody positive VKH patients were all female and it was statistically significant compared to anti-EsteD antibody negative VKH patients. There was no statistical difference in other examined factors. ^a^Student’s t test; ^b^Fisher’s exact probability test.
